# Modeling Epidemics in Seed Systems and Landscapes To Guide Management Strategies: The Case of Sweet Potato in Northern Uganda

**DOI:** 10.1094/PHYTO-03-18-0072-R

**Published:** 2019-08-13

**Authors:** K. F. Andersen, C. E. Buddenhagen, P. Rachkara, R. Gibson, S. Kalule, D. Phillips, K. A. Garrett

**Affiliations:** 1 Plant Pathology Department, University of Florida, Gainesville, FL 32611-0680, U.S.A; 2 Institute for Sustainable Food Systems, University of Florida, Gainesville, FL 32611-0680, U.S.A; 3 Emerging Pathogens Institute, University of Florida, Gainesville, FL 32611-0680, U.S.A; 4 Department of Rural Development and Agribusiness, Gulu University, Gulu, Uganda; 5 Natural Resource Institute, University of Greenwich, Greenwich, United

**Keywords:** disease control and pest management, ecology and epidemiology, postharvest pathology and mycotoxins, techniques, virology

## Abstract

Seed systems are critical for deployment of improved varieties but also can serve as major conduits for the spread of seedborne pathogens. As in many other epidemic systems, epidemic risk in seed systems often depends on the structure of networks of trade, social interactions, and landscape connectivity. In a case study, we evaluated the structure of an informal sweet potato seed system in the Gulu region of northern Uganda for its vulnerability to the spread of emerging epidemics and its utility for disseminating improved varieties. Seed transaction data were collected by surveying vine sellers weekly during the 2014 growing season. We combined data from these observed seed transactions with estimated dispersal risk based on village-to-village proximity to create a multilayer network or “supranetwork.” Both the inverse power law function and negative exponential function, common models for dispersal kernels, were evaluated in a sensitivity analysis/ uncertainty quantification across a range of parameters chosen to represent spread based on proximity in the landscape. In a set of simulation experiments, we modeled the introduction of a novel pathogen and evaluated the influence of spread parameters on the selection of villages for surveillance and management. We found that the starting position in the network was critical for epidemic progress and final epidemic outcomes, largely driven by node out-degree. The efficacy of node centrality measures was evaluated for utility in identifying villages in the network to manage and limit disease spread. Node degree often performed as well as other, more complicated centrality measures for the networks where village-to-village spread was modeled by the inverse power law, whereas betweenness centrality was often more effective for negative exponential dispersal. This analysis framework can be applied to provide recommendations for a wide variety of seed systems.

The identification of key locations for surveillance and management is an important problem in plant disease epidemiology, and it remains an open question whether the same locations are best for both in any given system. There are often multiple mechanisms for pathogen dispersal that must be integrated in models of dispersal risk to identify key locations. For example, formal and informal seed trade networks and the dispersal of pathogens by vectors may both be important risk components. Seed trade networks, or seed systems, are a critical component of global food security, but often they also serve as human-mediated pathways for the regional and global dispersal of plant pathogens.

Seed systems circulate planting material to farmers from a range of formal (commercial or regulated sector) and informal (farmer- based) sources (Coomes et al. 2015). Seed system actors may include farmers, multipliers, traders, non-governmental organizations (NGOs), seed companies, breeding organizations, and communities. Seed security—defined as timely access to quality planting material by all at a fair price (Almekinders et al. 1994; Gibson et al. 2011; McGuire and Sperling 2013; Sperling 2008)—is vital for improved livelihoods, particularly for smallholder farmers. Adequate access to diverse seed material with favorable traits, such as disease resistance or nutritional benefits (e.g., b-carotene biofortification), may also bring with it increased risk for novel pathogen introduction. Newly introduced pathogens or pathotypes can be particularly problematic, because methods for detection may be limited or unavailable, and host resistance may be unattainable for several years.

The provision of “clean” or “pathogen-free” seed is a major challenge to any seed system. In most low-income countries, this problem is pronounced, with seed provisions that are local and largely of unknown quality. A majority of farmers keep seed from previous seasons or obtain seed from neighbors, local traders, or local markets, with some instances of long-distance trade (Delaquis et al. 2018; Gildemacher et al. 2009; Pusadee et al. 2009). Having robust models of seed systems supports policy development, action planning in the face of emerging epidemics (such as surveillance, quarantine, variety deployment, and education), and risk assessments for possible system disruptions caused by shocks, such as climate change or political unrest.

A dramatic example of a likely seed-transmitted disease occurred in 2011 when maize lethal necrosis (MLN) was first reported in Kenya (Wangai et al. 2012) and soon was detected in several neighboring countries. MLN symptom expression results from coinfection with maize chlorotic mottle virus and a potyvirus (Mahuku et al. 2015). It is likely that at least maize chlorotic mottle virus was first introduced through infected seed and then spread rapidly through the landscape via seed and vector transmission. Since its introduction, MLN has been detected in several East African countries, including Ethiopia, Uganda, South Sudan, Tanzania,the Democratic Republic of Congo (DRC), and Rwanda (De Groote et al. 2016; Hilker et al. 2017; Mahuku et al. 2015). In our study, we consider an informal sweet potato seed system, where “seed” is not true botanical seed but vine cuttings. In vegetatively propagated seed systems, viruses and other seed-transmitted diseases are particularly important risks to yield and quality over successive cycles of propagation, and methods of control are limited (Thomas-Sharma et al. 2016). The problem of seed-transmitted virus introduction was illustrated in 2014 when Sri Lankan cassava mosaic virus (causing cassava mosaic disease) was first reported in Cambodia, presumably being introduced through infected seed material, and it has since been reported in Vietnam and China (Delaquis et al. 2018; Graziosi et al. 2016; Uke et al. 2018; Wang et al. 2016).

Network analysis is a powerful analytic tool for studying the role of system components used across many disciplines, including the analysis of epidemic spread and control in human, animal, plant, and computer systems (Buddenhagen et al. 2017; Keeling and Eames 2005; Pastor-Satorras and Vespignani 2001; Shaw and Pautasso 2014; Silk et al. 2017). Seed systems and other epidemics mediated by trade networks are particularly amenable to network analysis, because they are inherently networks with a suite of actors (network nodes) that move both genetic material and information through space and time (network links) (Pautasso 2015). Epidemics that are mediated by networks introduce underlying contact structures in contrast to simpler models that assume homogeneous mixing (Keeling and Eames 2005). Contact structures may be empirically observed or estimated by other means (Wiratsudakul et al. 2018). For example, connectivity may be estimated based on the distance between nodes in a landscape.

Our study builds on concepts previously developed for studying epidemic spread in large- and small-scale plant trade networks (Buddenhagen et al. 2017; Moslonka-Lefebvre et al. 2011; Nelson and Bone 2015; Pautasso 2015; Pautasso and Jeger 2008; Pautasso et al. 2010) by considering not only observed seed trade network data but also, estimated dispersal risk outside the formal trade network (Harwood et al. 2009; McQuaid et al. 2017). Previous studies of seed systems have often focused on understanding the effects of social ties and how well seed system networks may conserve variety diversity in the landscape (Abay et al. 2011; Garrett et al. 2017; Pautasso 2015; Pautasso et al. 2013). For example, Pautasso et al. (2013) found that the degree distribution of an Ethiopian barley seed network, and particularly the outdegree of the starting node, influenced the percentage of the network that could be reached by a new variety. In this study, we combined data layers for observed formal seed trade and inferred informal seed and vector movement in a multilayer network, which we use to model epidemic risk and in region- wide simulation experiments. Multilayer networks can be used to model integrated seed system components, such as the combination of seed movement networks and management information networks (Buddenhagen et al. 2017; Garrett 2018).

High-resolution data were collected for an informal sweet potato vine seller network, where the communities to which each seller sold material were recorded over the course of a season along with the relative quantities. However, spread of seed material and associated pathogens after the vines reached the communities was not studied and required estimation. When model parameters are unknown and difficult or impossible to observe, sensitivity analysis and uncertainty quantification can be used to evaluate the influence of parameter choice on model outcomes (Campolongo et al. 2000). In this study, we considered a range of values of the spread parameters of both the inverse power law and negative exponential models selected based on the assumption that there is a greater tendency for farmers to exchange planting material with neighboring villages than with those that are distant (Perales et al. 2005; Pusadee et al. 2009), and the probability of vector spread decreases with increasing distance. We evaluated the influence of parameter values on network link formation and subsequent epidemic progress under several scenarios of pathogen spread.

Network analysis can play an important role in evaluating nodes important for targeted surveillance and mitigation of the movement of pathogens or other contaminants through seed systems and landscapes (Sutrave et al. 2012). Node centrality statistics—such as node betweenness, eigenvector, and degree centrality—can be calculated to define key nodes and actors in a system and forecast the risk of pathogen introduction, pathogen spread, or technology diffusion in a cropping system (Garrett 2012; Garrett et al. 2018; Harwood et al. 2009; Moslonka- Lefebvre et al. 2011; Pautasso 2015; Pautasso and Jeger 2008; Sanatkar et al. 2015). For example, a study of wheat grain movement in the United States and Australia identified key locations that could be strategically targeted for sampling and management of mycotoxins (Hernandez Nopsa et al. 2015). For soybean rust in the United States, priorities for targeting geographic nodes for sampling to forecast epidemic movement were identified (Sanatkar et al. 2015; Sutrave et al. 2012). Although there are a number of statistics available that are likely to be associated with the importance of a node in an epidemic, it is an open question as to which statistics are most important for prioritizing nodes for monitoring and management in real-world networks (Holme 2017). The nodes that are most important for epidemic maximization, quarantine, or surveillance (important traits for epidemic mitigation) are not always the same nodes (Holme 2017), and the best criteria for choosing nodes may be specific to a given system (Holme 2018). In our simulation experiments, nodes were selected for management based on their network centrality measures, mimicking a real-world scenario where it is necessary to apply management technologies before complete information about an invasion. In our study, we define “management” as the restriction of the exchange of infected seed material; in practice, it is through phytosanitary regulation, quarantines, or the introduction of intensive management interventions.

In this study, we propose a general framework for analyzing multilayer networks of observed and estimated seed trade data that can be translated to a broad range of seed systems. We (i) analyze, as a case study, key network properties within areal-world seed system important to regional food security; (ii) evaluate variety dissemination within this observed network, comparing the distribution of landraces and higher-nutrient introduced varieties; (iii) model the progress of a potential seedborne pathogen introduced into the network in a series of simulation experiments and compare the use of different network statistics as selection criteria for nodes to be intensively managed, nodes that maximize epidemic spread, and nodes best for epidemic surveillance; and (iv) perform sensitivity analysis/uncertainty quantification to determine the sensitivity of outcomes to the spread parameters of the models (both the inverse power law and negative exponential) used to construct the village- to-village transaction networks.

## Materials And Methods

**Study system: Sweet potato in northern Uganda**. This study examines sweet potato vine transactions in northern Uganda. Sweet potato is a major staple food crop in many African countries, and Uganda is the second largest producer in Africa (fourth globally) (FAOSTAT 2013). Sweet potato is generally grown by women in Uganda in small plots of land close to the home, and it is important for household food security (Behrman 2011; Johnson and Gurr 2016). In the last decade, sweet potato has increased in importance because of the introduction of β-carotene biofortified, orange-fleshed sweet potatoes (OFSP) by HarvestPlus, part of the CGIAR Research Program on Agriculture for Nutrition and Health. OFSP varieties were introduced with the goal of addressing vitamin A deficiency in women and children in this region (Behrman 2011). The infusion of improved varieties through these programs along with increasing adoption (Obong et al. 2017) could potentially serve as a new source of risk for disease spread in the region, which is another reason why we highlight OFSP dissemination in this study.

Viral diseases are biotic constraints to sweet potato production in Uganda and throughout sub-Saharan Africa, with the most yield- limiting being sweet potato virus disease (SPVD), which occurs when a plant is coinfected with sweet potato feathery mottle virus (SPFMV) and sweet potato chlorotic stunt virus (SPCSV) (Karyeija et al. 1998). Seed degeneration is the successive loss in yield over generations of seed planting material owing to the accumulation of viruses and other seedborne pathogens, a particularly important problem for vegetatively propagated crops (Thomas-Sharma et al. 2016). Degeneration is highly problematic in informal seed systems where farmers tend to save seed season to season and where certified seed sources are rare or nonexistent. The normal planting material for sweet potato is foliar vine cuttings, and both SPFMV and SPCSV can be transmitted to succeeding generations this way, with evidence of yield degeneration over five generations in high- pressure fields in Uganda (Adikini et al. 2015). SPVD has not yet been reported in northern Uganda, likely because the extended dry season in this region is often unfavorable for the whitefly vector (R. Gibson, personal observation). Changes in climate patterns or vector range, however, could potentially extend the range of this disease into this region. The prospect of novel pathogen introduction makes it important to model epidemic scenarios to inform intervention strategies.

**Fig.1:**
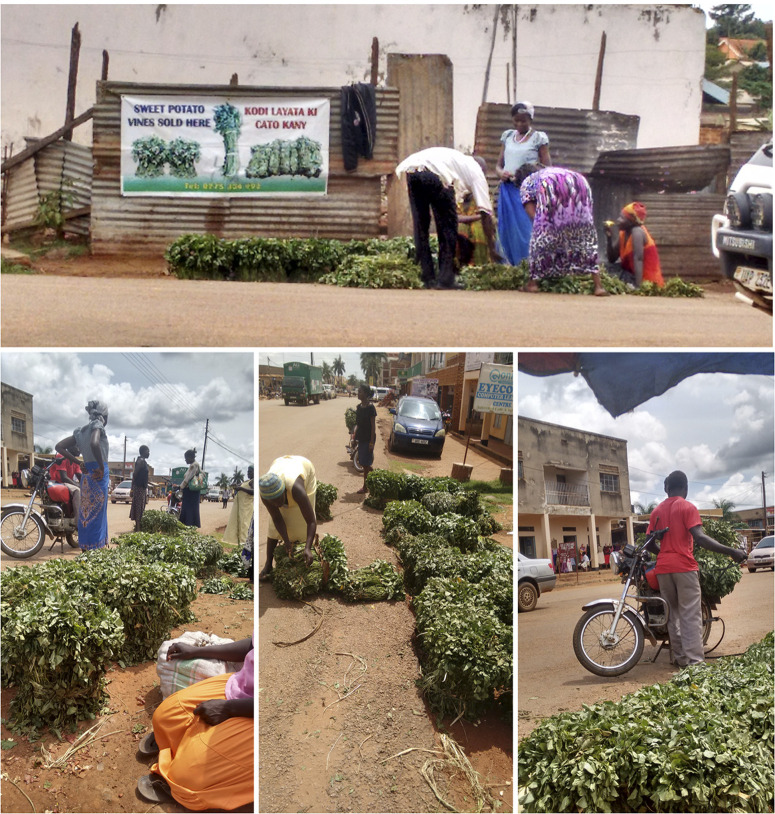
Sellers in the Gulu region of northern Uganda selling sweet potato vines in 2014. Photo credit: Paul Rachkara.

In northern Uganda, sweet potato seed material is sold in small bundles of vine cuttings (Fig.1). Multiplication is typically carried out by smallholder farmers who have access to a limited number of fields with adequate moisture to produce roots and vines through the extended dry season, which typically lasts from December to April (Gibson 2013). This is followed by distribution of vine cuttings via local markets and at farm gate. These offseason multipliers generally produce local landraces, which tend to be well-adapted white-fleshed cultivars (Gibson 2013). Vine cuttings are not easily stored, and because of the single extended dry season in northern Uganda, vines need to be obtained by farmers at the beginning of each season (Gibson et al. 2011). There are also several formal institutions involved in sweet potato breeding and distribution in Uganda, including the National Sweetpotato Program, the International Potato Center, private sector enterprises, and NGOs (Gibson 2013).

**Survey methods.** A survey of vine multipliers and sellers was conducted in 2013 and 2014 in the Gulu region of northern Uganda; it is fully described by Rachkara et al. (2017). A more complete cohort of multipliers and sellers was surveyed in 2014, the focus of our analysis. In this system, most multipliers are also vine sellers, and therefore we simplify our terminology to refer to surveyed individuals as sellers. Each seller was visited weekly from the start of the growing season (April) and surveyed twice per week to record all transactions (purchases and sales) that occurred in the period since they were last visited until the end of the season (August). Volume of transaction (number of bundles), price, variety, origin of buyer, and buyer type (farmer or seller) were recorded. In this study, a small bundle refers to 50 vines cut to 40 cm in length. Large bundles are equal to 20 small bundles. Because of the high volume of transactions, names of individual buyers were not collected, and therefore, sales transactions were summarized by the buyer’s village.

**Fig.2:**
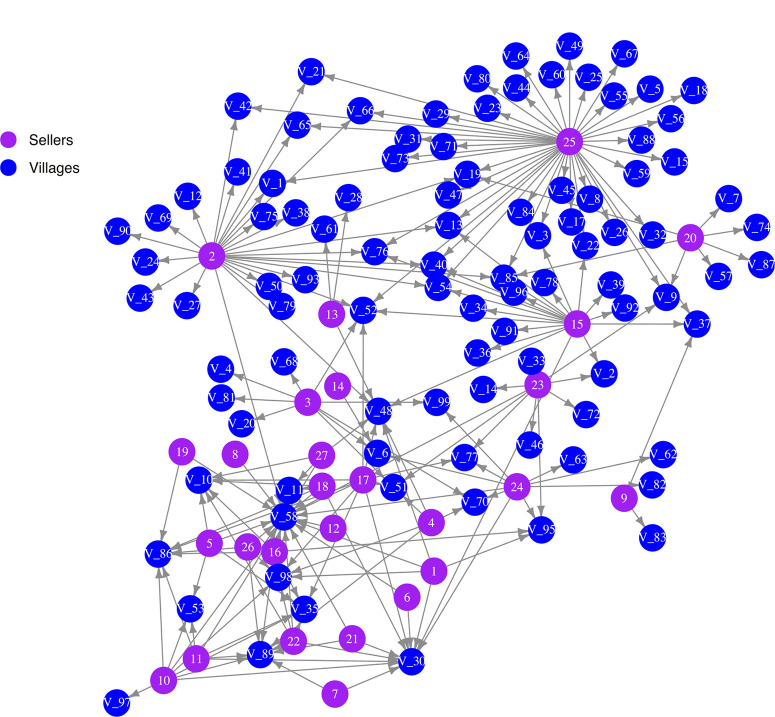
Network structure of sweet potato vine transactions reported in the 2014 growing season in northern Uganda with both sellers (lighter nodes) and villages (darker nodes). Links represent the occurrence of at least one transaction in the 2014 growing season. The graph layout was generated with the Davidson–Harel layout algorithm and does not represent the geographic locations of villages.

**Seed network analysis.** Nodes in this analysis include sellers (*n* = 27) and villages (*n* = 97), with one set of directed links representing vine sales from an individual seller to an individual village (a bipartite network)(Fig. 2). Only villages that customers in the survey reported as their farm locations were included in analyses. Villages in this region of Uganda typically are composed of 40 to 60 households. Although several transactions were recorded on a weekly basis, transactions were aggregated for this analysis so that seller-to-village links represent the existence of at least one transaction over the course of the season. Seller-village links were based entirely on the data from Rachkara et al. (2017). In total, the seller-village transaction network consisted of 124 nodes (27 sellers and 97 villages). Network node statistics for this bipartite network, such as node strength, node degree, and degree distribution, were calculated using the igraph package (Csardi and Nepusz 2006) in the R programing environment (R Core Team 2018).

**A sensitivity analysis of the spread parameter describing village-to-village spread**. It is important to not only understand the movement of seed material from vine sellers to villages but also to understand the potential movement of seed material or vectors between villages (farm to farm). This type of geospatial spread data was unavailable; therefore, links between villages were estimated as a function of distance, and the uncertainty of the model outputs was quantified. We assumed based on conventional knowledge of informal seed systems and studies that have evaluated movement of crop genotypes in similar systems (Delaquis et al. 2018; Labeyrie et al. 2016) that movement of seed or vectors would be more likely between villages that are more proximate, still allowing for a nonzero probability of long-distance movement.

The probability of village-to-village spread was modeled using an inverse power law model and a negative exponential model, with results compared in a sensitivity/uncertainty analysis. The sensitivity of epidemic outcomes (methods described in detail below) along with the efficacy of management treatments were compared for each dispersal kernel. The set of parameters was selected to represent a range of potential pathogens and patterns of informal seed exchange, capturing less common instances of long-distance connections. The inverse power law equation used here was y = *d*^- β^ , where y is the probability of movement between the villages, *d* is the distance (meters) between a pair of villages, and β is the spread parameter (Jongejans et al. 2014). The negative exponential function was exp(-λ*d*), where λ is the spread parameter. The matrix of distances (meters) between villages in this study was calculated using the Haversine formula (Sinnott 1984) implemented using the R package geosphere (Hijmans 2017). The minimum distance between villages in this dataset was 100 m, and the maximum was 411,436 m. An uncertainty analysis was performed to determine the influence of the parameters β and λ (Table 1)on link formation and the resulting epidemic simulation outcomes. A range of values for both β and λ was selected, bounded by parameter values that produce a full network (all possible links) and a near-zero probability of link formation (Supplementary Figs. S1 and S2).

**Fig. 3:**
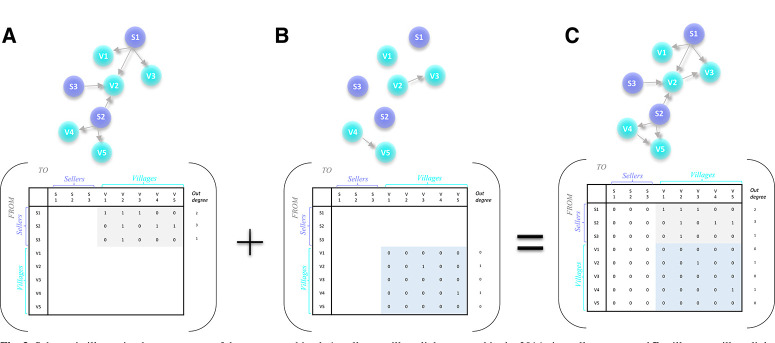
Schematic illustrating how two types of data were combined: A, seller-to-village links reported in the 2014 vine seller survey and B, village-to-village links estimated based on the distance between villages using an inverse power law or negative exponential model of the probability of movement. The schematic represents a hypothetical network of three sellers and five villages, each represented as a node in the network. A link between nodes is represented as a one in the matrix, and absence of a link is indicated by a zero. Matrices were combined in C, a –supranetwork— with both seller-to-village and village-to-village links. Note that, in this study, all potential seller-to-seller links and village-to-seller links were set to zero, with no transactions taking place in this direction. A “supranetwork” based on the Ugandan sweet potato data was used in the simulation experiments presented in this study.

For each parameter combination and time step, in each simulation (additional details about epidemic simulations are described in the following section), the set of village-to-village links was stochastically generated using one of the above described dispersal kernels (inverse power law or negative exponential), and village-to-village links were combined with observed transaction links to form an expanded adjacency matrix or “supraadjacency matrix” (Fig. 3). A link in the supranetwork represents a seed movement event (transaction) or movement of potentially viruliferous vectors (but not necessarily an infection event, which is then driven by the probability of transmission).

It is important to note that only villages of farmers who purchased vines from sellers surveyed in this study were included in this analysis, and there may be villages in this region that contribute to the risk of epidemic spread but were not included. Because of this, estimates of the performance of management may be biased upward .

Network properties, such as the number of nodes and network density, were calculated for the 2014 season supranetwork. Node measures, such as degree, strength, PageRank, and betweenness centrality, were evaluated for both villages and sellers (Table 2).

**Modeling epidemic spread in simulation experiments**. Utilizing the above-described supranetwork (including both estimated village-to-village and observed seller-to-village links), we performed simulation experiments with corresponding parametric sensitivity analyses to address the following questions. (i) What are the optimum risk-based surveillance locations for pathogen monitoring if there is equal likelihood of pathogen introduction at each node? (ii) What effect does the seller (and seller node degree) through whom disease is introduced have on disease progress and final disease outcome? (iii) How much can disease spread be limited by implementing intensive management interventions, where managed nodes (villages) cannot become infected or spread disease? (iv) How do network centrality statistics compare in terms of their utility for selecting intensive management locations?

In each experiment, simulations were conducted over 20 time steps and repeated 500 times. The epidemic model was a discrete time network susceptible-infected Markov chain. The probability *P*_*i,t*+1_ that node i either remains infected or becomes newly infected in discrete time is defined as

$$p_{i,t+1}=p_{it}+\left(1-p_{it}\right)\left(1-q_{it}\right)$$

q_*it*_ is the probability that node *i* does not become infected in time *t*,

$$q_{it}=\prod_{j\neq i}\left(1-\varphi y_{ij}p_{jt}\right)$$

where φ is the probability of infection transmission (on an existing link) and y_ij_ is the probability that a link exists between nodes i and j. For the seller-to-village portion of the adjacency matrix, y_ij_ is based on the observed transactions, and for the village-to-village links, it is based on the dispersal kernel (negative exponential or inverse power law). The probability of pathogen transmission (φ) was fixed to 0.01 for each realization. P_it_ for t = 1 is a special case for the initial conditions, and it varies from one experiment to another as described in detail below. Because recovery does not occur, the diagonals of the adjacency matrix were set to one. Sellers, other than the starting seller, cannot become infected during a realization (epidemic spread is illustrated in Supplementary). Simulations were carried out using custom R code (https:// garrettlab.com/ugsweets).

**Experiment One: The value of villages as risk-based surveillance locations**. We evaluated the importance of each village as a risk-based pathogen surveillance location: that is, how important the location is for monitoring new pathogen introductions into the region. Risk-based surveillance takes into account the role of sampling locations in epidemics (Cameron 2012; Parnell et al 2014; Stark et al. 2006). In this scenario analysis, we evaluated all pairwise combinations with one node (seller or village node) as the starting point for epidemic introduction and another node being monitored for infection. The outcome for each node being monitored was the number of other nodes that remained uninfected when the node being monitored became infected, as in Buddenhagen et al. (2017) and the INA R package (https://garrettlab.com/software) (Garrett 2018). The higher the number of uninfected nodes when the disease is detected at the surveillance location, the more options for regional disease management remain, and therefore, the surveillance location is more useful. In this analysis, we compare both sellers and villages for their relative value for epidemic monitoring. Over the 500 realizations, the mean and variance of the number of nodes uninfected by the time that the pathogen reached each node were calculated. Each node (both village and seller) was then assigned a “risk-based surveillance score” defined as the mean percentage of nodes uninfected by the time that the pathogen is present in each potential surveillance node across all starting nodes. In this analysis a higher value of the risk- based surveillance score indicates a better surveillance location, because more nodes remain uninfected at detection. (Note that this is a modification in output format compared to the analysis in Buddenhagen et al. (2017), where a lower score indicated fewer nodes infected and thus a lower score was preferable.) Summarizing over all of the potential starting nodes allowed for comparison of the importance of each village as a potential location for surveillance for an introduced pathogen when each potential starting location was considered equally likely. Sensitivity analyses were incorporated in this experiment to determine the influence of the dispersal kernel parameters (driving village-to-village link formation) on the risk-based surveillance score. We repeated the simulation for networks built from each of the range of parameter values (β and λ) tested for both the inverse power law and the negative exponential models. This sensitivity analysis represents scenarios where community exchange events range from very frequent to extremely rare. Pairwise Spearman’s rank correlation was calculated for all parameter combinations to evaluate how consistent the ranking of the village risk-based surveillance scores was across parameter values.

**Table 1:** Parameters describing dispersal probabilities in a seed system network

Parameter	Definition	Values	Comments
β	Exponent of the power law, where β>0	0.0001–0.9 (0.0001, 0.01, 0.1, 0.2, 0.3,^a^ 0.4,^a^0.5,^a^ 0.6, 0.7, 0.8, 0.9)	Larger values of β result in steeper declines in the probability of movement with distance and thus, fewer village-to-village links
λ	Scale parameter of the exponential model	0.000001–0.002 (0.0000001, 0.00001,^a^0.00005,^a^ 0.0001,^a^ 0.001,^a^ 0.002)	Larger values of λ result in fewer village-tovillage links; probability of a link declines exponentially with distance
φ	Probability of transmission of infection at each time step over an established link from an infected node to a susceptible node	0.1^a^	Applied to both seller-to-village and village-to-village links

a Value that was used as the default in simulation experiments.

**Experiment Two: Identifying epidemic maximizers**. The second simulation experiment evaluated potential spread through the seed network, where each of the 27 surveyed sellers was evaluated as the starting point of the epidemic. (Exclusion of village nodes as potential starting points was in contrast to the above scenario, where each node was equally like to be the starting point.) Evaluating each seller across parameter values allows us to determine if there are features of particular starting nodes, such as node degree, that would lead to an increased or decreased potential for disease spread in the system.

In this experiment, at the start of each realization (time 1 of 20), the “starting seller” was assigned an infection status of one, and all others were set to zero. The starting seller maintained infection status throughout the realizations, and all other sellers remained uninfected. Epidemic simulations were conducted as previously described. Villages that became “infected” after one time step maintained infected status in the subsequent time step (time t + 1) and thus, could infect villages to which they had links with the same probability (φ = 0.01) in the subsequent time step (time t + 2). Once infected, villages remained infected and infectious throughout the course of the time series. The number of villages infected was evaluated across 20 time steps in 500 realizations across the 11 parameter values of the inverse power law and six parameter values of the negative exponential dispersal kernels to evaluate the frequency distribution of outcomes. To evaluate the differences in disease progress between starting nodes, the area under the disease progress curve (AUDPC) was calculated by summing the trapezoids between time steps under the curve (Madden et al. 2007).

**Experiment Three: The influence of intensive management on infection dynamics**. This experiment analyzed the value of five approaches for identifying locations for intensive management in advance of an epidemic introduction. The approaches were based on selection of locations according to their (i) betweenness centrality, (ii) PageRank centrality, (iii) degree centrality, and (iv) risk-based surveillance score, as well as (v) at random (details below). We evaluated the influence of intensive management treatments on disease progress in the network over time. Intensive management was defined here as the removal of the potential for a node to transmit or acquire infection in each time step, with selected nodes maintaining this managed status from time 0 to time 20 (also known as node “quarantine” or “immunization” in epidemic network literature). Intensive management in this scenario potentially represents the common practical scenario of phytosanitary quarantine by regulatory agencies after the detection of pathogens in new regions or the implementation of a wide-scale development effort to limit pathogen transmission between localities. Although complete removal is a simplification, this allows for exploration of the maximum level of influence of treatments while allowing the analysis to focus on the effects of the network topologies on treatment efficacy and epidemic spread. All sellers were equally likely to be the starting point for infection, with the starting seller drawn at random at the start of each simulation.

The influence on epidemics of applying intensive management to 0 to 97 (or 0 to 100% of) nodes was evaluated (Supplementary). The effect of management treatments was evaluated across four representative values of the spread parameter β (0.3, 0.4, 0.5, and 0.6) in the inverse power law model used to estimate village-to-village spread (Supplementary Table S1) and four representative values of λ (1e-5, 5e-5, 1e-4, and 1e-3) in the negative exponential model (Supplementary Table S2). Nodes (villages only) for management were selected based on their rank for each of the five methods in simulations implemented over 20 time steps in 1,000 realizations. The AUDPC was calculated for each management treatment across values of β and λ based on the percentage of nodes infected at each time step (excluding those selected for management).

We performed the above management analysis with villages selected based on their rank for node betweenness centrality (Freeman 1979), PageRank centrality (Brin and Page 1998), and degree centrality in comparison with a scenario where villages were drawn at random (Table 2). We also compared the utility of these key network node statistics (Table 2) with the use of the previously calculated risk- based surveillance score (calculated in experiment 1). Node centrality measures (node degree, betweenness, and PageRank centrality) were calculated by taking the mean of each of these centrality measures for 1,000 networks generated stochastically for each of the values of the spread parameters (β and λ).

## Results

**General network properties**. In 2014, 27 sellers were tracked, resulting in a total of 878 individual vine sales to farmers from 97 distinct villages in Uganda. The seller-to-village portion of the adjacency matrix was estimated based on aggregated transactions from sellers to villages (Fig. 2). This network has a total of 124 nodes and 204 links (link density = 0.013), with a degree distribution that approximately follows a power law or scale-free distribution (Supplementary Fig. S5). The addition of estimated village-to-village links increased the number of links as a function of the parameter chosen for the dispersal kernel. The number of links roughly represents the number of potential vine transactions or movement of vectors in each time step in simulation experiments. Node degree, or the number of incoming (indegree) and outgoing (outdegree) links, was calculated for each seller node (mean = 7.6, minimum = 1, and maximum = 42). Node degree was positively correlated with node strength, or the sum of all of a seller’s transactions (Pearson’s correlation coefficient = 0.86) (Supplementary Fig. S6). Node degree, betweenness centrality, and PageRank centrality were also measured for all villages in this analysis (Table 2) and used in subsequent analyses.

**Table 2:** Network node centrality measures and relevance for epidemics in seed systems

Measure	Definition	Relevance for epidemics in seed systems
Degree centrality	The number of links that a node has to other nodes in the network (both incoming and outgoing)	Node degree of epidemic starting point (number of links) has been shown to influence epidemic outcomes; those with high degree may be “superspreaders” once infected
Betweenness centrality	The number of shortest paths through the network of which a node is a part (Freeman 1979)	A measure of how much a node serves as a “bridge” to new nodes; removal of nodes with high betweenness may contain an epidemic within a region
PageRank centrality	A special case of eigenvector centrality, also known as the Google algorithm; a weighted sum reflecting both direct links to a node (degree) and the node degree of neighbors. Suitable for directed networks (Brin and Page 1998)	If a node does not itself have a high node degree but is connected to nodes with high degree, it may still be at increased risk of infection and spreading infection, and high-degree nodes may be even more important if they have high-degree neighbors
Risk-based surveillance score	The number (or percentage) of nodes that remain uninfected at the time when infection occurs at the node in question (Buddenhagen et al. 2017; Garrett 2018)	A higher score indicates greater utility for surveillance at a node being considered (but not necessarily greater utility for management at that node)

**Fig.4:**
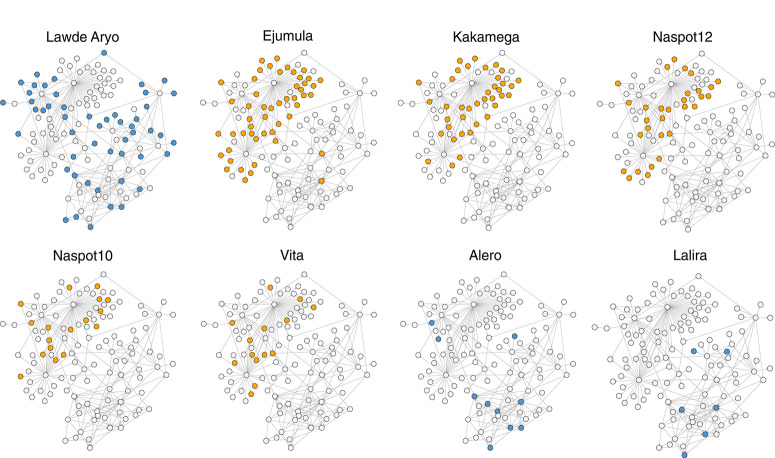
Networks of dissemination of the top eight cultivars sold during the 2014 growing season in terms of number of transactions reported. All sellers and villages surveyed are represented in each network, whereas filled nodes represent sellers and villages involved in the sale or purchase of the specified cultivar. Unfilled villages did not access a given variety in 2014 through this surveyed seed network. Node shade indicates white-fleshed landraces (darker) and orangefleshed sweet potato (lighter) cultivars.

**Variety dissemination**. Fifteen cultivars were sold during the 2014 season (Supplementary Fig. S7), including landraces (all white fleshed) and cultivars introduced by the national breeding program. Six of these cultivars were OFSP cultivars, and they were disseminated by only two sellers in many individual transactions. By comparison, the most common white-fleshed landrace, cultivar Ladwe Aryo, was sold by 25 distinct sellers in hundreds of transactions throughout the season. When the network is examined separately by variety, disaggregation becomes apparent (Fig. 4). Although both Ladwe Aryo and the OFSP cultivar Ejumula reached 51 villages each, only eight of these villages purchased both (Fig. 4). Evaluating networks representing the distribution of the top eight varieties to villages in the network (Fig. 4) indicates that only a small number of sellers and villages were purchasing orange-fleshed varieties from the sellers surveyed. It seems from this survey that most individuals from a single village only bought a single variety, even when they had access to multiple sellers.

**Fig.5:**
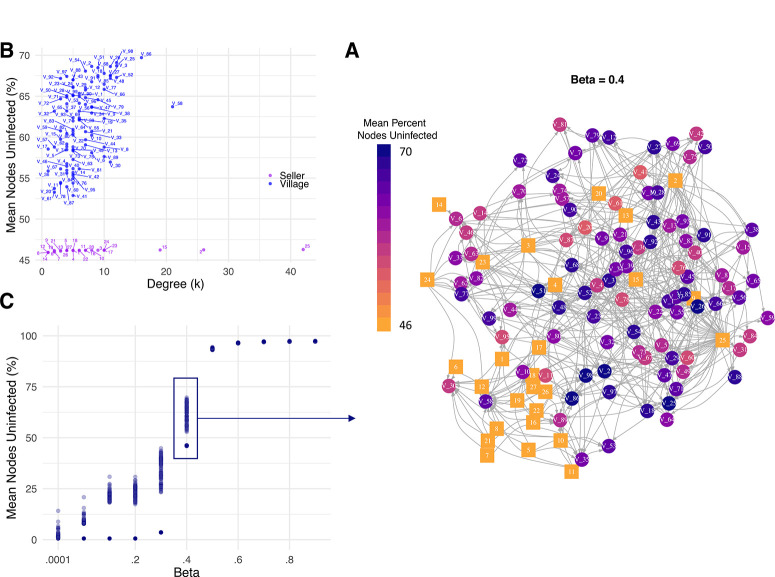
For the scenario where each node was an equally likely starting point for an epidemic, we evaluated the value of each village based on a “risk-based surveillance score”, or mean percentage of nodes still uninfected by the time that the pathogen reaches that node in simulations. Higher values indicate better potential for detecting the pathogen at this location before the colonization of the remainder of the network. A, Network constructed from the transaction matrix and estimated with the inverse power law model with an intermediate value of the spread parameter β(0.4). The network has a total of 124 nodes (97 villages and 27 sellers) with 380 links (204 from sellers to villages and 176 between villages). Circles represent villages, and squares represent sellers. The graph layout is based on the Davidson–Harel layout algorithm, which locates nodes with links closer together and those without links farther away according to a simulated annealing algorithm (implemented in igraph). B, Individual node degree (sum of incoming and outgoing links) against “mean percentage of villages uninfected,” with seller nodes lighter and village nodes darker, for β = 0.4. C, The “mean percentage of nodes uninfected” for each node for each of 11 values of the spread parameter β evaluated. β = 0.4 is highlighted to indicate that it has been visualized in the network graph. Additional graphs for the values of β and network illustrations for other β values are in Supplementary Video S1.

**Experiment One: The value of villages as risk-based surveillance locations**. For the scenario where each node was an equally likely starting point for an epidemic, we evaluated the value of each village for surveillance and assigned each a risk-based surveillance score. A village was considered a more effective surveillance location if a large proportion of other nodes was still uninfected when the pathogen reached that village. We repeated this experiment across 11 values of β and six values of λ, the spread parameters used to drive the inverse power law and negative exponential models of link formation between villages, respectively. For the inverse power law model, we found that, at low and high values of the spread parameters, surveillance scores were similar for all nodes (Fig. 5C), likely owing to very high or very low network connectivity at these extremes. Nodes differed in their value for surveillance primarily at intermediate values of the parameters. For a reference value of β = 0.4 (corresponding to approximately two links per village), the mean percentage of nodes still uninfected by the time that the pathogen reached any given node ranged from 46 to 70% (Fig. 5A). Higher values are desirable, because they indicate that the pathogen will be detected at this node when a relatively high number of villages remain uninfected for more efficient monitoring. Sellers had the lowest values, and village values ranged from 51 to 70% (Fig. 5B). The village with the highest risk-based surveillance score (70%) was V_86 (i.e., having the highest mean number of villages uninfected by the time the pathogen would be present at that village)(Fig. 5A). Interestingly, these nodes also had a relatively high average node degree (Fig. 5B), and overall, for power law parameter β = 0.4, the risk- based surveillance score was positively correlated with node degree (Spearman’s rank correlation coefficient = 0.33, P value = 0.0002).

As β increased, the number of village-to-village links in the network decreased and the risk-based surveillance score increased across all nodes (Fig. 5C). The lower values of the spread parameter greatly decrease the probability of link formation between villages, dramatically limiting disease spread so that sellers and their links become the biggest drivers of epidemic spread, and thus, their value for surveillance increases slightly (Supplementary Video S1). However, as β decreased, more links were added between villages, and thus, infection levels increased (Fig. 5C). When the spread parameter is very low (β = 0.0001), nodes all tended to have similar utility as surveillance locations, because the pathogen rapidly colonized the network in most scenarios. Prioritizing nodes based on a risk- based surveillance score will be most useful when there is intermediate exchange between communities in any given season.

**Fig.6:**
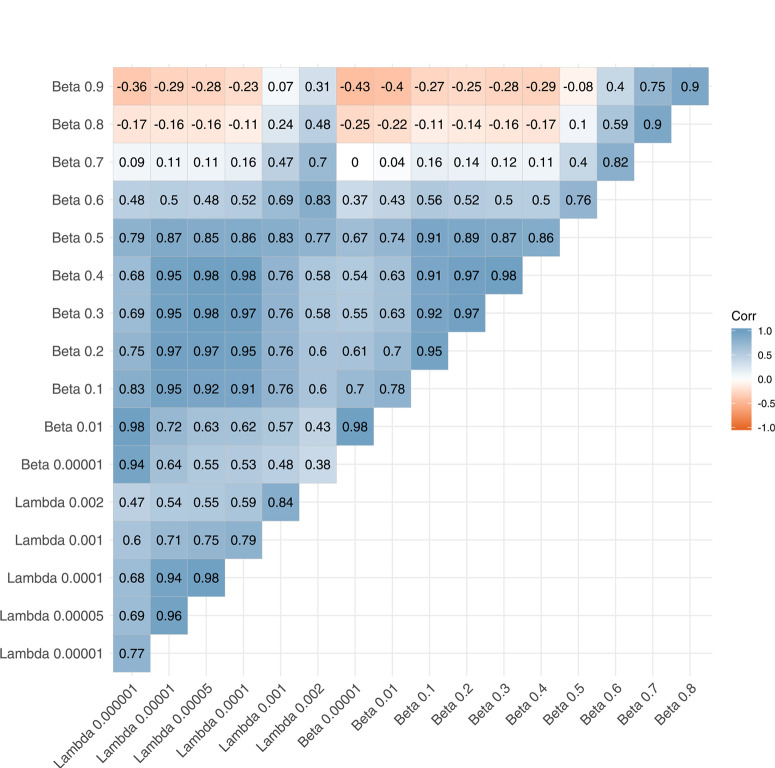
Spearman's rank correlation for risk-based surveillance scores across parameter values for spread parameters λ (negative exponential) and β (inverse power law).

To assess the influence of the model chosen for village-to- village links, this experiment was also conducted for networks estimated for six values of the spread parameter λ of the negative exponential model (Supplementary Figs. S8 and S9 and Supplementary Video S2). Although the number of links generated varied across parameter values and as a function of the kernel chosen to model spread, the rank of nodes was highly correlated based on Spearman’s rank correlation coefficient (Fig. 6). Correlations were particularly high for intermediate spread parameter values and lower at the extremes, indicating that the main model outcome (the selection of important locations to monitor) may not be sensitive to the parameter chosen provided that the parameter value is intermediate.

**Fig.7:**
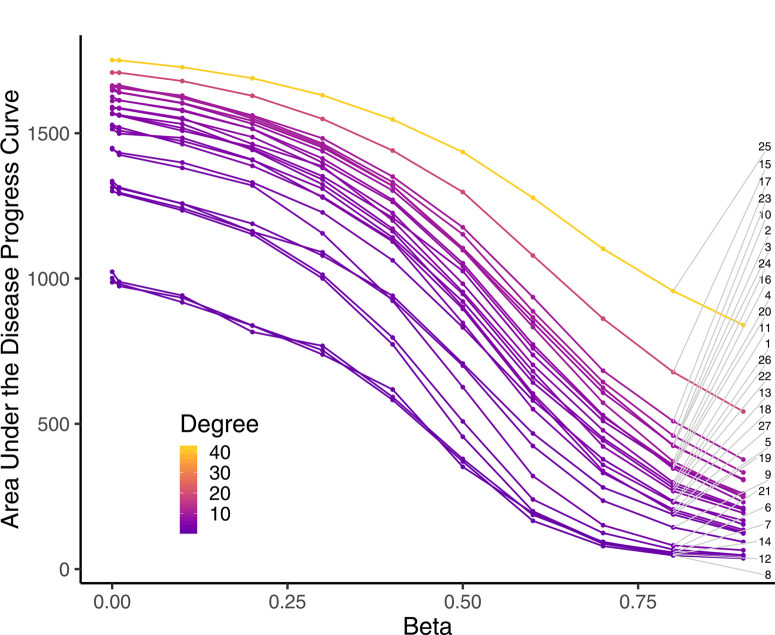
Model sensitivity to the spread parameter β from the inverse power law model. The graph shows pointwise mean area under the disease progress curve (AUDPC) for disease progress over 20 time steps over 11 values of β. Each line indicates 1 of the 27 sellers (seller identification numbers labeled in gray) who was the starting point for disease in the network. Subsequent disease progress is through the network of villages in the landscape. Each point represents the AUDPC for 500 realizations of disease spread through the network. Line color represents node degree (total number of incoming and outgoing links) for each seller.

**Experiment Two: Modeling epidemic progress as a function of starting seller**. All 27 sellers were compared as starting nodes for an introduced epidemic simulated over 20 time steps in 500 realizations, and results were evaluated across the parameter ranges of β (Fig. 7) and λ (Supplementary Fig. S10). Mean AUDPCs for infections starting with each seller were compared across parameter values based on seller node degree Fig. 7. Seller node degree ranged from 1 to 42, with sellers with the lowest node degree (sellers 8, 14, and 12; node degree = 1) having the lowest mean AUDPC across parameters for each dispersal kernel. Similarly, the seller nodes with the highest node degrees (seller 25; node degree = 24 and seller 15; node degree = 19) had the highest AUDPC values across parameter values (for node 25, β = 0.00001; mean AUDPC = 1,751 and β = 0.9, mean AUDPC = 840). AUDPC declined with increasing β (decreasing links) across all nodes, in which case the starting node seemed to be the most important driver of epidemic progress. There was an exception, however, at intermediate values of b, where it seems that network topology (not starting node) influenced disease progress more than at the extreme parameter values (Fig.7). A similar trend was observed for the six parameter values of λ.

**Experiment Three: The influence of management on infection dynamics**. This experiment was conducted to evaluate the utility of particular villages based on common network centrality statistics for preventative intensive management in the case of a novel pathogen introduction into the region. We evaluated the influence of intensively managing villages, which here is a simplified scenario where villages cannot become infected or transmit infections to other villages in the network. Nodes were selected based on node degree rank, betweenness centrality, PageRank centrality, and the risk-based surveillance score (calculated in the first experiment). Mean node degrees for the 40 nodes selected as management candidates ranged from 5.0 to 13.7, from 1.5 to 6.0, from 0.6 to 2.8, and from 0.2 to 1.4 (with the highest selected first) for each value of β (0.3, 0.4, 0.5, and 0.6, respectively). Mean betweenness centrality values ranged from 53.2 to 331.2, from 31.8 to 433.4, from 0.3 to 18.5, and from 0.0 to 0.9 and mean PageRank values ranged from 0.0 to 0.013, from 0.0 to 0.01, from 0.01 to 0.01, and from 0.01 to 0.01 for each value of b, respectively. Generally, the rank of nodes was correlated across β values (Supplementary Fig.S11).

**Fig.8:**
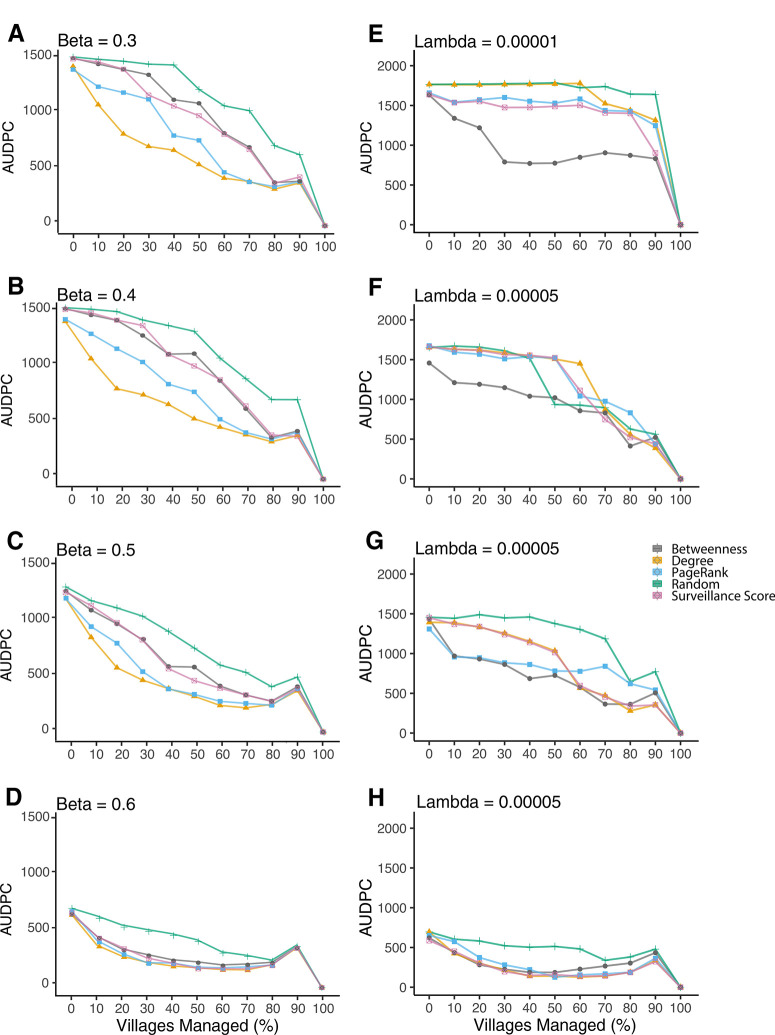
Sensitivity analysis results across four intermediate values of the spread parameters, for both the A to D, inverse power law and E to H, negative exponential models of link formation. Mean AUDPC values were calculated across five scenarios where 0 to 100% of villages were intensively managed, in increments of 10. Managed nodes were selected based on five methods: node betweenness centrality, degree centrality, PageRank centrality, surveillance score (calculated in experiment 1), and random selection. Each point represents mean AUDPC based on the percentage of nodes infected out of those not quarantined over the course of 20 time steps across 1,000 realizations. There were 97 villages total in the system and 27 sellers. The seller that served as the starting point for the epidemic in each simulation was drawn at random. The slight increase in mean AUDPC for the case where 90% of nodes are quarantined is a function of the small number of nodes (;10) remaining with the potential for infection. When these do become infected, it leads to a larger percentage of potential nodes infected (illustrated in Supplementary Fig. S15). Values for the treatment where 100% of nodes were quarantined, although undefined, are represented as zero.

Across each value of β, node degree was the most effective criterion for removing nodes to slow epidemic progress compared with betweenness centrality, PageRank centrality, risk-based surveillance score, and random selection of villages. For β = 0.3, AUDPC values decreased by 11, 40, and 56% when 10, 40, and 70 nodes were managed, respectively, compared with no management. A similar trend was found for β = 0.4, 0.5, and 0.6 (Fig. 8 and Supplementary Fig. S12). It was not surprising that the “smart” selection criteria (centrality measures and surveillance score) almost always outperformed the “naive” selection criteria (randomly selecting villages to manage) (Fig. 8). The effects of management were more pronounced with increasing β (Fig. 8), indicating that containment of an infection may become more difficult as the number of links in the network increases. As β increases (β = 0.4 and 0.5), PageRank centrality also greatly limits disease spread across 0 to 90% of managed nodes, although using node degree still slowed disease progress as well as or better than other centrality measures. At β = 0.6, all methods except randomly selecting nodes perform similarly, indicating that the fewer links, the easier it is to control an outbreak.

Interestingly, the best method for node selection was betweenness centrality across values of λ for networks generated using the negative exponential model (Fig. 8 and Supplementary Fig. S13). This is likely driven by the “thin-tailed” feature of the exponentially bound kernel, which results in exponentially decreasing likelihood of a link with distance, making the nodes with high betweenness centrality key for managing an outbreak. Across values of λ, each of the “smart” selection criteria outperforms the treatments where nodes were selected randomly for management Fig. 8. Degree centrality, betweenness centrality, Page Rank centrality, and the risk-based surveillance score were positively correlated across values of λ (Supplementary).

## Discussion

This analysis addressed practical questions in network epidemiology of which nodes are best for risk-based pathogen surveillance, intensive management, and epidemic maximization and if they are one in the same (Holme 2017; Radicchi and Castellano 2017). Our findings are consistent with analyses of exact small graphs (Holme 2017) and real-world networks (Radicchi and Castellano 2017), which suggest that optimal locations for each of these purposes may be different. This is an important consideration for management of plant disease epidemics in landscapes where there are simultaneous efforts to monitor pathogen introduction and to reduce spread.

The question of the optimal surveillance locations in a region is critical if there is any chance for mobilization of management tactics at a landscape level. Villages in the Gulu region of northern Uganda that were included in this study were identified as potential risk-based surveillance targets. Selection was based on how frequently the pathogen would be detected in these locations before substantial parts of the rest of the network become colonized in simulations. This surveillance score (Buddenhagen et al. 2017; Garrett et al. 2018) calculated for villages was highly correlated across intermediate spread parameters when comparing the parameter range both within and between the two dispersal models (both the negative exponential and inverse power law models). The insensitivity of surveillance node selection to dispersal parameter values is an important feature for its potential application across seed systems, suggesting that the exact number of links between locations is not critical for identifying the best locations for surveillance. The ability to generalize selection criteria for surveillance across parameter values is an important finding for informal seed systems, where data are often unavailable, and deserves future attention to evaluate the translation of this method across pathosystems. The identification of risk-based surveillance locations in the landscape can complement field-based diagnostic technologies, such as loop-mediated amplification assays (Yasuhara-Bell et al. 2016) or smartphone-assisted crop disease image detection (Mohanty et al. 2016), which are becoming increasingly available to practitioners and have the potential for rapid onsite detection of viruses and other pathogens. The method used here, applied to a region in northern Uganda, can also support pathogen surveillance efforts on a national or greater scale.

To answer the question of which nodes serve as “epidemic maximizers,” we identified sellers with the highest potential for disease spread in the network if they served as the introduction point for an epidemic (with infected planting material). We found in experiment two that the node outdegree of the “starting seller” (or the seller with infected planting material) seemed to drive the severity of epidemic progress, similar to the trend observed on hypothetical small networks (Pautasso et al. 2010). Interestingly, the influence of these high-degree sellers, who also were the main sellers of OSFP varieties, remained constant across the two dispersal kernels tested for link formation (the negative exponential model and the inverse power law model) and across a suite of spread parameters evaluated for each in a sensitivity analysis. Our findings expand on previous studies indicating that the epidemic starting point influences both epidemic progress and epidemic outcome (Pautasso 2015; Pautasso et al. 2010). The epidemic maximizers may also be variety adoption maximizers, taking into account both seed sales and potential village-to-village spread of new varieties based on village proximity.

In this system, we compared several node centrality measures for their utility to limit epidemic spread with intensive management. Centrality measures are important indicators of risk within epidemic networks (Banks et al. 2015; Holme 2017; Kiss et al. 2006). In this case, management was represented as the complete quarantine or immunization of a node in the network. We found that, across a range of scenarios for the power law model of dispersal, node degree centrality performed as well as or better than centrality measures that take into account the broader network topology (PageRank centrality, betweenness centrality, or risk-based surveillance score) and always better than a random selection of nodes. This finding of comparable or better control with management targets selected based on node degree is important for the mitigation of invasive pathogens in plant exchange networks, because node degree is one of the simplest centrality measures to collect and calculate (Christley et al. 2005). Furthermore, this confirms findings from human epidemiology literature (Lloyd-Smith et al. 2005) in large-scale networks, where node degree has been shown to be comparable with more complicated centrality measures for the selection of nodes to be immunized (Salathe et al. 2010). We found that, when the village-to-village dispersal was modeled by the negative exponential, however, betweenness performed as the best choice for mitigating epidemic spread. Node betweenness is based on the average number of shortest paths in which a node is included and may prove to be a better option when spread is primarily local or on small-world networks (Holme et al. 2002). Centrality measures have also been explored in animal trade networks, where centrality measures (such as degree) have been significantly associated with on-farm disease levels and disease progress (Kiss et al. 2006; Lee et al. 2017; Salines et al. 2018), and deserve additional attention for plant disease epidemics in landscapes.

As a general strategy, in times of epidemic emergence, high-degree locations are good candidates to be targeted for control strategies, such as quarantine in the form of phytosanitary regulation. Similar methods to those described here may be used to target villages for development projects that aim to disseminate resistant varieties to key hubs and maximize both the spread of disease resistance genes and the impact of these genes on epidemics. The presence of resistant sweet potato varieties in the landscape has been associated with a decrease in virus incidence (Aritua et al. 1999). However, betweenness centrality may prove to be more important for identifying key nodes in some other types of seed systems or landscapes in which there are many nearly separate modules with a small number of links between them. In such seed systems, the nodes with high betweenness centrality that bridge these modules might be particularly important for surveillance or quarantine.

Although management treatments were effective at slowing epidemic progress when a large percentage of nodes was included, here we found that management was not sufficient for halting epidemic spread under any scenario (when <100% of nodes were quarantined). This property of rapid disease spread is consistent with other scale-free networks, where high-degree “superspreaders” can rapidly transmit disease to other nodes in the network in a small number of steps (Banks et al. 2015; Jeger et al. 2007; Lloyd-Smith et al. 2005). Rapid spread is also a property that is consistent with other plant disease epidemics where dispersal includes low- probability long-distance dispersal events (Mundt et al. 2009; Severns et al. 2019).

Future research not only should aim to assess the efficacy of management introduction at key nodes, but also may include economic considerations. Cost-effectiveness has been studied, for example, for large-scale disease reduction strategies in sub-Saharan African maize (Wu and Khlangwiset 2010). Economic considerations may dictate optimal thresholds for phytosanitary policy in seed systems, where standards that are too high do not allow for production and standards that are too low allow for extensive disease spread (Choudhury et al. 2017). There is also the potential for policies to integrate risk owing to network structures and risk owing to climate disease conduciveness for establishing phytosa- nitary thresholds. Previous models have included both the costs and benefits of disease control on hypothetical small-world and local lattice networks (Kleczkowski et al. 2011), a method that could be extended to this framework by adding terms for the cost of implementing large-scale interventions to control plant disease along with the potential benefits. In the case of OFSP, benefits would be in terms of household food security, household profit, and health outcomes. Future research should also consider the influence of the deployment of other components of an integrated seed health strategy (Thomas-Sharma et al. 2017) into models for epidemic mitigation, such as positive selection, resistant varieties, clean seed, and education about disease progress and management strategies. Integrated seed health strategies may be easier and more cost effective to deploy than strict phytosanitary restrictions or introducing complete “quarantine” of villages in these systems, where social associations are major drivers of exchange between villages and access to certified clean planting material may be minimal to nonexistent.

It is important to note that this study was based on data limited to sellers who participated in weekly monitoring in the Gulu region of northern Uganda. Although there was an intensive effort to identify sellers, there may have been other sellers or sources of vines that were not captured. In addition, there are other villages with sweet potato fields in the region of study that could possibly be sources of disease, but they were not included in this analysis, because they were not associated with a buyer in the dataset. In the model used here, after nodes become infected, they remain infected (and infectious) through the duration of the time course. Because there may be options for epidemic recovery in this sweet potato seed system (through reversion, roguing, or positive selection), future studies may include a recovery term in the model. Also, in our model, disease presence is taken to coincide with disease detection; however, a lag in detection would be a useful feature to explore in a future model. Another outstanding question is the influence of multiple simultaneous starting points on epidemic progress and outcomes in seed systems.

Along with epidemic potential, the analysis of network topologies presented here shows how sales of OFSP varieties and landrace varieties differed. When vine distribution was disaggregated by variety, it was clear that most cultivars were not well disseminated by seller-to-village links (Fig. 4). We found that a single white- fleshed landrace variety, Ladwe Aryo, dominated landrace sales in this season, whereas a single OFSP vine, Ejumula, dominated OFSP sales (Fig. 4). Interestingly, there is little overlap between the villages where farmers bought these varieties. Based solely on the observed sales, we cannot determine whether it was preference or availability of planting material of OFSP varieties that drove sales, or a combination of the two. This distinction is important, however, because much effort has been made to promote improved OFSP varieties (Low et al. 2017). In a study in 2015 (Obong et al. 2017), 89% of 51 multiplier fields sampled in the Gulu region during the 2015 offseason grew local landraces, with the remainder of area planted to improved varieties. This proportionally low rate of adoption might be attributed to the introduction of OFSPs into the region (with promotion by HarvestPlus and affiliated NGOs), and adoption may increase in coming years if these varieties become accepted by farmers and if there remains an adequate local supply of vines provided by these informal sellers. Seed exchange is often closely related to kinship ties, language, and social organization patterns (Abizaid et al. 2016; Labeyrie et al. 2016; Perales et al. 2005; Westengen et al. 2014), and future research to better model the influence of social structure on variety diffusion in this seed system network could inform strategies to increase OFSP adoption. Useful future studies could assess the rate of adoption over time in the landscape to determine the implications of the models of spread that we present for each type of variety.

There is a tradeoff in the effects of high centrality in seed networks. That is, it is favorable to be a village with high degree (many links) because of the increased availability of a diversity of cultivars (like OFSP varieties) from more sellers. However, high levels of connectivity can make a village more susceptible to pathogen invasion, and they increase its likelihood of serving as an epidemic “superspreader” as illustrated by our management experiments, where after high-degree villages were managed, the epidemic was successfully reduced.

Understanding the dynamics of epidemics in seed systems is critical for effective pathogen monitoring, risk assessment, and epidemic management (Buddenhagen et al. 2017; Garrett et al. 2018; Harwood et al. 2009; Shaw and Pautasso 2014). Although some plant disease literature explores this topic, research on epidemics in real-world seed networks is still in an early stage. Future surveys that include questions about social ties and the movement of information among farmers will support better models of variety adoption and distribution in seed systems. Next research steps will include more finely parameterizing transmission patterns, including the impact of variety resistance and vector biology, and modeling system adaptation to sustained exogenous shocks and stressors. There is the potential to include data about known yield degeneration rates and known environmental conditions (Thomas-Sharma et al. 2017) to predict regional yield loss in the case of pathogen introduction. One of the challenges for developing strategies for epidemic management in seed systems may be a tendency for each seed system to have different optimization requirements (Holme 2018; Kleczkowski et al. 2011). The framework that we present here can be applied to seed systems in general to evaluate nodes key to the spread of new varieties and nodes key to epidemic surveillance and management.
